# Potential Zoonotic Origins of SARS-CoV-2 and Insights for Preventing Future Pandemics Through One Health Approach

**DOI:** 10.7759/cureus.8932

**Published:** 2020-06-30

**Authors:** Muralikrishna Konda, Balasunder Dodda, Venu Madhav Konala, Srikanth Naramala, Sreedhar Adapa

**Affiliations:** 1 Veterinary Sciences, Banfield Pet Hospital, Flower Mound, USA; 2 Veterinary Medicine, Holiday Park Animal Hospital, Pittsburgh, USA; 3 Hematology and Oncology, Ashland Bellefonte Cancer Center, Ashland, USA; 4 Hematology and Oncology, King's Daughters Medical Center, Ashland, USA; 5 Rheumatology, Adventist Medical Center, Hanford, USA; 6 Nephrology, Kaweah Delta Medical Center, Visalia, USA

**Keywords:** coronavirus disease 2019, sars-cov-2, sars-cov, zoonosis, reverse zoonosis, one health approach

## Abstract

Coronavirus disease 2019 (COVID-19) is an emerging infectious disease that has resulted in a global pandemic and is caused by severe acute respiratory syndrome coronavirus-2 (SARS-CoV-2). Zoonotic diseases are infections that are transmitted from animals to humans. COVID-19 caused by SARS-CoV-2 most likely originated in bats and transmitted to humans through a possible intermediate host. Based on published research so far, pangolins are considered the most likely intermediate hosts. Further studies are needed on different wild animal species, including pangolins that are sold at the same wet market or similar wet markets before concluding pangolins as definitive intermediate hosts. SARS-CoV-2 is capable of reverse zoonosis as well. Additional research is needed to understand the pathogenicity of the virus, especially in companion animals, modes of transmission, incubation period, contagious period, and zoonotic potential. Interdisciplinary one health approach handles these mosaic issues of emerging threats by integrating professionals from multiple disciplines like human medicine, veterinary medicine, environmental health, and social sciences. Given that the future outbreak of zoonotic diseases is inevitable, importance must be given for swift identification of the pathogen, source, and transmission methods. Countries should invest in identifying the hot spots for the origin of zoonotic diseases, enhance diagnostic capabilities, and rapid containment measures at local, regional, and national levels. The threat posed by emerging infectious diseases in modern-days also needs combined efforts internationally where a single discipline or nation cannot handle the burden alone.

## Introduction and background

Coronavirus disease 2019 (COVID-19) caused by severe acute respiratory syndrome coronavirus 2 (SARS-CoV-2) is spreading at an unrelenting pace worldwide, and World Health Organization (WHO) declared it as global pandemic [1]. As of June 15th, 2020, globally, 7.96 million COVID-19 cases were confirmed, and this has resulted in 434,388 deaths [2]. COVID-19 pandemic is a grave reminder of the imminent global threat from emerging infectious diseases. Emerging infectious diseases are defined as infections that have newly surfaced in a population or have existed but are swiftly increasing in incidence [3]. In the 100 plus years that have passed since the 1918 Spanish flu pandemic, which was the worst natural catastrophe of the twentieth century, COVID-19 stands out as a pandemic that approaches its magnitude by any measure [4, 5]. As demonstrated by COVID-19, under suitable circumstances, a newly emerging infectious disease originating anywhere in the world could spread to all continents within weeks to months [6].

The outbreak of COVID-19 was initially linked to a local seafood market in Wuhan, China, where the sale of wild animals has been implicated as the primary source of SARS-CoV-2 infection [7]. SARS-CoV-2 is 79.6% identical to severe acute respiratory syndrome coronavirus (SARS-CoV), the virus that caused the SARS epidemic of 2002-2003, and 96.2% similar to a bat coronavirus RaTG13 (BatCoV RaTG13) at the whole genome level [8]. Based on the viral genome sequence and evolutionary analysis, Chinese horseshoe bats of genus Rhinolophus have been considered as a natural reservoir host for the SARS-CoV-2 virus [8-10]. However, as of now, no definitive intermediate host was found. Based on the isolation of a closely related genome from Malayan pangolins (Manis javanica), they are thought to be intermediate hosts of the SARS-CoV-2 [11-13]. Therefore, definitive identification of intermediate host(s) is highly informative in elucidating the exact mechanisms of origin of the SARS-CoV-2 virus.

The SARS-CoV, epidemic of 2002-2003 is thought to have passed from bats to humans through other animals sold at the wet markets in Guangdong, China, such as Himalayan palm civets, Chinese ferret badgers, and raccoon dogs [14]. Similarly, the direct or indirect spillover of SARS-CoV-2 from animals in or near the wet food markets of Wuhan, China, is implicated as the primary source of SARS-CoV-2 infection. However, definitive identification of the animal host as a source of SARS-CoV-2 requires the detection of the virus in situ in an infected animal at the source of the initial outbreak [7]. In the absence of this crucial piece of the puzzle, a detailed review of current knowledge of the role of all animals as a source of primary infection or re-infection of SARS-CoV-2 to humans is imperative. Review and discussion of this information potentially help in stemming the ongoing outbreak and enhance global preparedness against similar future emerging zoonotic diseases.

## Review

Zoonoses

According to the Centers for Disease Control and Prevention (CDC), about 75% of emerging infectious diseases originate in animals [15]. Approximately 60% of known infectious diseases in people, such as rabies, ringworm, and salmonellosis, are transmitted from animals. Animals thus play a vital role in maintaining infectious agents in nature [16]. These diseases are known as zoonotic diseases or zoonoses. Zoonoses can be viral, bacterial, parasitic, or may involve unconventional agents such as prions as in bovine spongiform encephalitis. A zoonotic disease is an infection that is naturally transmissible from vertebrate animals to humans as per the WHO [17]. Zoonotic diseases can cause human and economic losses. The World Bank estimated that between 2001-2010, zoonotic diseases have a direct cost of more than $20 billion, with over $200 billion in direct losses to the economy worldwide [18]. There is a widespread agreement among economists that the economic damage from the COVID-19 pandemic has severe negative impacts on the global economy. Notably, emerging infectious diseases put a strain on the health systems, and particularly the countries with limited health care resources will get impacted because they have limited capacity for diagnostics and infection control. These factors stress the importance of streamlining animal health surveillance systems, and the vital role they play in anticipating, detecting, and containing future zoonotic disease outbreaks [19]. 

In the past two decades, there have been three coronavirus outbreaks, the SARS-CoV (2002-2003), the MERS (2012-2013), and SARS-CoV-2 (2019). The primary source of origin of all these novel coronaviruses in people appears to be from animal to human transmission. After attaining inter-human transmission, all these coronaviruses cause acute respiratory illnesses with varying degrees of infectivity and pathogenicity. Based on the current understanding SARS-CoV-2 appears to be more effective in inter-human transmission and less pathogenic as compared to SARS-CoV and MERS-CoV. Overview of the similarities and differences of SARS-CoV-2 from other related coronaviruses helps in elucidating the mechanistic differences in their infectivity and pathogenicity [20].

Coronaviruses

Coronaviruses (CoVs) are named after crown-like (corona in Latin) spike projections from the virus membrane when observed under an electron microscope [21]. All coronaviruses are classified as a subfamily Orthocoronavirinae within the Coronaviridae family. Based on phylogenic clustering, the Orthocoronavirinae subfamily has been sorted into four genera, the alpha, beta, gamma, and delta coronaviruses. All of the seven know human CoVs (HCoV) are in two of these genera: alpha coronaviruses (HCoV-229E, and HCoV-NL63), and beta coronaviruses (HCoV-HKU1, HCoV-OC43, MERS-CoV, SARS-CoV, and SARS-CoV-2) [22]. In animals and humans, coronaviruses can cause respiratory, enteric, and central nervous system diseases. Through mutations and recombination that occur with relative ease, they are capable of adapting to new environments. Hence, coronaviruses are programmed to alter host range and tissue tropism efficiently [23].

Coronaviruses are large enveloped viruses with a genome of ~30 kilobase positive-sense single-stranded RNA. The replicase gene coding for the non-structural proteins occupies about two-thirds of this genome at 5′ end; the remaining one-third of viral genome codes for the structural and accessory proteins. Typically, the coronaviral genome contains genes coding for four structural proteins, namely, the spike (S), membrane (M), envelope (E), and nucleocapsid (N) proteins. The genes for accessory proteins are interspersed within the structural genes at the 3′ end of the genome. The S protein is a homotrimeric glycoprotein that makes up the distinctive spikes of the virion and mediates attachment of the virus to the host receptor. In most coronaviruses, S protein is cleaved by a host cell protease into two separate polypeptides, S1 and S2. The S1 polypeptide makes up the large receptor-binding domain (RBD) of the S protein, while the S2 forms the stalk of the spike molecule [23].

In general, the host range of coronaviruses is very narrow. The ability of a coronavirus to replicate in a given cell type depends solely on the ability of virion to interact with its host receptors [24]. The S protein is one of the best-characterized proteins in coronaviruses like SARS-CoV, MERS-CoV, and SARS-CoV-2. Based on the findings so far, S protein is believed to play a crucial role in overcoming species barriers and accomplishing interspecies transmission from animals to humans [24, 25]. Coronavirus, with the aid of their RBD on S1 polypeptide, recognizes a multitude of host receptors who have their own physiological functions. For example, both SARS-CoV and SARS-CoV-2 recognize a zinc peptidase angiotensin-converting enzyme 2 (ACE2), whereas MERS-CoV recognizes a serine peptidase namely dipeptidyl peptidase 4. The S2 polypeptide of S protein that makes up fusion peptide fuses viral and host membranes [25-27]. After entry into the host cells, the viral replicase coding region is first translated to yield the polyproteins, which are subsequently cleaved by two viral proteases to yield non-structural proteins essential for viral replication. The remaining genome codes for the structural proteins of the virus. Based on the phylogenic analysis and evolutionary studies, most zoonotic novel coronaviruses have shown to be originated from bats and have been reported to be transmitted to humans by aerosols thorough intermediate hosts infected by the virus [28]. 

Potential origin of SARS-CoV-2

Huanan seafood market in Wuhan city, Hebei Province, China is linked to early cases of COVID-19. As reported by the China CDC, 33 out of 585 environmental samples from the Wuhan Seafood markets showed evidence of SARS-CoV-2 [29]. These samples were mostly from the market's western portion, where wildlife was sold. As of now, there is no scientific evidence on the detection and isolation of SARS-CoV-2 from the seafood market apart from the China CDC statement. Specifically, there are no published reports about samples collected from wildlife in the market. There were still no scientific reports about patient zero in China, which could have aided in tracking the definitive origin of SARS-CoV-2. SARS-CoV-2 most likely originated in Rhinolophus affinis bat species based on 96.2% genomic homology between SARS-CoV-2 and BatCoV RaTG13 [8]. Despite their genomic similarity, BatCoV RaTG13 diverges at RBD, which suggests that it may not bind efficiently with human ACE2 and could have evolved in a different animal species before infecting humans [30]. Previous investigations of SARS-CoV and MERS-CoV origins concluded that Himalayan palm civets [14] and dromedary camels [31] acted as intermediate hosts. There is a possible intermediate host involvement in the transmission of SARS-CoV-2 as well. Unless a definitive intermediate host is identified, there are high risks for new outbreaks of SARS-CoV-2 like viruses. Furthermore, exploring SARS-CoV-2 intermediate host(s) will also help conduct investigations to evaluate the host-pathogen relationship and disease dynamics [32].

There have been initial hypotheses that snakes [33] or turtles [34] can be possible intermediate hosts for SARS‐CoV‐2. Luan J et al. disproved these theories based on the composition of ACE2 in these animals and their differences with human ACE2 [35]. They also implied that simulated structures indicated ACE2 proteins from animals of Bovidae and Cricetidae families were able to associate with RBD of SARS-CoV-2 and should be included in the screening of intermediate hosts. Based on the modeling of RBD of SARS-CoV-2 and palm civet ACE2 interactions, palm civets were less likely to be the intermediate hosts. SARS-CoV-2 is less likely to replicate efficiently in rats and mice, ruling them out as intermediate hosts [30]. Wan Y et al. also proposed that pigs, ferrets, cats, and non-human primates might serve as intermediate hosts for SARS-CoV-2 [30]. Additionally, SARS-CoV-2 is known to cause permissive infections in ferrets and cats, implying the possibility of other animals as potential reservoirs or intermediate hosts [36, 37]. There has also been speculation that pangolins can act as possible intermediate hosts. The Malayan pangolins are predominantly found in South East Asian countries. Poaching and illegal transportation of these endangered species have been reported throughout Asia. Viral genomic analyses of dead pangolins from the Guangdong wildlife rescue center revealed that two out of the 11 pangolins carried viral genomes belonging to SARS-CoV-like coronaviruses [38]. Reinvestigation of the published data through metagenomic analyses from dead Malayan pangolin lung samples showed genomic and evolutionary evidence of a SARS-CoV-2-like virus. Coronavirus isolated from pangolins (pangolin-CoV) is identical to SARS-CoV-2 by 91.02% and to BatCoV RaTG13 by 90.55% at the whole genome level. Moreover, the S1 protein of the pangolin-CoV is more closely related to SARS-CoV-2 than BatCoV RaTG13. Specifically, the five key amino acid residues in the RBD involved in the interaction with human ACE2 receptors are entirely consistent between pangolin-CoV and SARS-CoV-2 [11].

Lam TTY et al. reported the presence of two sub-lineages of CoVs in pangolins that were seized in Southern China [12]. One of the sub-lineages is very closely related to SARS-CoV-2 at the receptor-binding domain. Another study by Xiao K et al. reported that one of the coronaviruses isolated from Malayan pangolins showed 100%, 98.2%, 96.7%, and 90.4% amino acid identity with SARS-CoV-2 in the E, M, N, and S genes, respectively [13]. This study also confirmed the RBD of the S protein of the pangolin-CoV is identical to that of SARS-CoV-2 except for one amino acid. Based on the comparison of available genomes, they suggested that SARS-CoV-2 might have originated from the recombination of a BatCoVRaTG13-like virus with a pangolin-CoV-like virus. Furthermore, Wong MC et al. also proposed that SARS-CoV-2 has originated in horseshoe bats but most likely evolved in pangolins based on a higher sequence identity at S1 that aids with host infection [39]. Li et al. also demonstrated that the receptor-binding motif of SARS-CoV-2 was introduced through recombination with pangolin coronaviruses, which appears to be a critical step in its ability to infect humans [40].

While current studies speculate that Malayan pangolins are most likely intermediate hosts [11, 12], other animals still can serve as intermediate hosts. The genome similarity of SARS and MERS CoVs is 99.8% [14] and 99.5% [31] to that found in civet cats and dromedary camels, respectively. Even though RBD of pangolin-CoV and SARS-CoV-2 are structurally identical, they are only 91% identical at the whole genome. This still leaves the question if pangolins are truly intermediate hosts or just incidental hosts [11, 13]. Li et al. also concluded that SARS-CoV-2 did not come directly from pangolins [40]. This conclusion is based on the finding that pangolin-CoV lacking the Arg-Arg-Ala-Arg (RRAR) motif between the boundary of S1 and S2 subunits that may have been involved in proteolytic cleavage of the S protein. More importantly, none of the pangolins sampled were from the Wuhan or nearby wet markets. Therefore, more studies are needed on different wild animal species, including pangolins that are sold at the same wet market or similar wet markets before concluding pangolins as definitive intermediate hosts.

Reverse zoonosis

Based on in-vivo studies, SARS-CoV-2 can replicate well in both upper and lower respiratory tracts of cats and ferrets [36, 37]. SARS-CoV-2 infection can also be transmitted between cats through droplets [36] and in ferrets through direct contact [37]. The presence of SARS-CoV-2 antibodies in cats from Wuhan during the COVID-19 outbreak is further proof that cats can get infected from humans or the environment [41]. As per Zhang Q et al., fifteen percent of the tested cats were positive for RBD of SARS-CoV-2 based indirect enzyme-linked immunosorbent assay (ELISA), and all the samples collected before the outbreak tested negative [41]. Dogs appear to be less susceptible to SARS-CoV-2 infection, and the virus replicates poorly in pigs, chickens, and ducks [36].

There are also reports of two dogs, two cats, four tigers, and three lions testing positive for SARS-CoV-2 [42]. These animals were presumed to have gotten the infection from their caretakers. Based on these findings, SARS-CoV-2 has the potential of reverse zoonosis (transmission from humans to animals) as well. Even though reverse zoonosis has been reported in a handful of cases, extensive testing of samples submitted for respiratory illnesses in cats, dogs, and horses did not yield any positive results [43]. The main drawback of this study is the lack of information if the tested animals were exposed to people infected with COVID-19. More studies are needed to understand the extent of reverse zoonosis, and if the virus from infected companion animals in its native/mutated form is capable of zoonosis. Extensive surveillance for the presence of viral RNA/antibodies in all domestic animals, at-risk wildlife species, and ways to contain SARS-CoV-2 or related viruses in affected animals are needed.

One health approach

Emerging infectious diseases can create multiple threats to humanity, and COVID-19 is a perfect example. Interdisciplinary one health approach handles these mosaic issues of emerging threats by integrating professionals from various disciplines like human medicine, veterinary medicine, environmental health, and social sciences. One health approach correlates the interconnections between people, animals, and the environment, thereby strengthening cooperative endeavors to improve the health of people and animals, including pets, livestock, and wildlife [44]. Notably, the collaborative one health approach globally is instrumental in mitigating the threat of emerging infectious diseases like COVID-19.

When an outbreak happens, several aspects need to be analyzed and understood like origin, native hosts, intermediate hosts, mutational tendency, transmission characteristics between animals-to-humans, and humans-to-humans. Mainly, reducing the risk of transmission from the natural hosts to humans include a thorough understanding of the natural ecology of the pathogens [32]. Given that bats are the primary source for the majority of the latest epidemics, understanding the habitats and diversity of bat species as reservoir hosts for coronaviruses and multiple other viruses is vital. Additionally, extensive research on bat physiology and immunology is critical in understanding how bats can harbor so many viruses without the clinical disease [45]. There is also a need to study the representative virologic biomes of all domestic and wild animals that have the potential to come in contact with humans. Importantly, all possible one health measures should be taken to minimize the interactions between bats, other high-risk wild animals, and humans.

Understanding the mode of transmission between natural hosts and their intermediate hosts is also another critical step to prevent future epidemics [32]. Identification of an intermediate host(s), and removing them from the wet markets is very critical. Removal of palm civets from wet markets prevented further epidemics of SARS after the initial outbreak [46]. Therefore, addressing live animal markets, in general, can play a vital role in preventing zoonotic transmissions [47]. This might include closing the markets if possible or at least limiting them to mixing very few species, separating live animals from processed meat, removing intermediate hosts if established, and rigorously testing for zoonotic pathogens. However, there are essential cultural facets involved with these markets, and social sciences play an indispensable role in changing these practices. Additionally, local governmental agencies should be involved in implementing stringent laws to limit the trade of endangered species. 

Extensive surveillance for SARS-CoV-2 should be done in all animals, including domestic livestock, companion animals, and wild animals, as they can be potential reservoirs or intermediate hosts, permit viral transmission among themselves, and can generate new viral strains [32]. Genetic analysis of SARS-CoV-2 along with other CoVs (SARS-CoV, MERS-CoV) in the past to identify the intermediate host is one of the significant one health measures. Once the origins of SARS-CoV-2 are established, the next step is to identify the appropriate measures to attenuate the transmission. Failure to identify and establish the intermediate host in the disease cycle can cause future outbreaks. Additionally, identifying intermediate hosts for SARS-CoV-2 also helps to understand the disease dynamics, host-pathogen interactions, and the possibility of reverse zoonosis [32].

Understanding the mode of transmission early in the disease cycle is very important. If the inter-human transmission is the reason for the rapid spread of disease, corrective measures such as practicing social distancing, using personal protective equipment, and avoiding unnecessary travel to limit the transmission might play a vital role in containing the outbreak. The public should also be educated on measures like self-quarantining when exposed to a person with a positive disease and seeking medical attention when they develop symptoms of the disease. The adoption of principles from one health approach helps in effectively coordinating these complex multidisciplinary tasks.

## Conclusions

COVID-19 reminds us of the failure of systems and safeguards that should have been in place to prevent a global pandemic of this magnitude. According to researchers, zoonotic diseases will continue to cross the animal-human barrier due to global population growth, destruction of animal habitats, urbanization, and consumption of traditional and non-traditional meats. Precisely, crowded conditions, mixing of multiple species, and human exploitation of endangered species as currently happening in wet food markets may have played a significant role in the origin of SARS-CoV-2. The highly contagious nature of the SARS-CoV-2 virus coupled with the geographical location of Wuhan (central China, which is the travel hub), and timing of the year (Lunar New Year - when many people travel nationally and internationally) led to the fast and uncontrolled global spread of the virus, which has never happened before in human history. The COVID-19 pandemic seems to be only the beginning of more possible disease outbreaks that are going to arise in the future. Further research is needed to understand the pathogenicity of the virus in companion animals, modes of transmission, incubation period, contagious period, and zoonotic potential.

Given that future outbreaks are inevitable, importance must be given for swift identification of the pathogen, source, and transmission methods. Countries should invest in identifying the hot spots for the origin of zoonotic diseases, enhance diagnostic capabilities, and rapid containment measures at local, regional, and national levels. Once the mode of transmission is identified, contact tracking systems should be in place to identify and curtail the further spread of the infection. Furthermore, the threat posed by emerging infectious diseases in modern-days needs combined efforts internationally where a single discipline or nation cannot handle the burden alone. There should also be a mechanism for dissemination of information, data sharing, and transparency among all countries when an outbreak happens. To face the future outbreaks and address human-animal-environmental interactions, there is a dire need for implementing multidisciplinary one health approach with international governmental cooperation.
